# Manganese in PET imaging: Opportunities and challenges

**DOI:** 10.1002/jlcr.3754

**Published:** 2019-07-11

**Authors:** Marie Brandt, Jens Cardinale, Ivo Rausch, Thomas L. Mindt

**Affiliations:** ^1^ Ludwig Boltzmann Institute Applied Diagnostics General Hospital of Vienna Vienna Austria; ^2^ Department of Biomedical Imaging and Image Guided Therapy, Division of Nuclear Medicine Medical University of Vienna Vienna Austria; ^3^ Center for Medical Physics and Biomedical Engineering Medical University Vienna Vienna Austria; ^4^ Department of Inorganic Chemistry, Faculty of Chemistry University of Vienna Vienna Austria

**Keywords:** cell labelling, immunoPET, manganese‐52g, PET/MRI, radiolabelled liposomes

## Abstract

Several radionuclides of the transition metal manganese are known and accessible. Three of them, ^51^Mn, ^52m^Mn, and ^52g^Mn, are positron emitters that are potentially interesting for positron emission tomography (PET) applications and, thus, have caught the interest of the radiochemical/radiopharmaceutical and nuclear medicine communities. This mini‐review provides an overview of the production routes and physical properties of these radionuclides. For medical imaging, the focus is on the longer‐living ^52g^Mn and its application for the radiolabelling of molecules and other entities exhibiting long biological half‐lives, the imaging of manganese‐dependent biological processes, and the development of bimodal PET/magnetic resonance imaging (MRI) probes in combination with paramagnetic ^nat^Mn as a contrast agent.

## 1 INTRODUCTION

Radioactive nuclides have been used in nuclear medicine for the assessment of functional processes since about a century.^1^ However, to be suitable for *in vivo* imaging applications, a radionuclide needs to meet specific physical demands: Its decay radiation should be in an energy range sufficiently high to escape a patient's body in detectable amounts but also low enough to allow an efficient measurement by available detectors. Suitable γ energies for this purpose are usually in the range of 50 to 600 keV for single photon emission computed tomography (SPECT) and 511 keV for positron emission tomography (PET). Furthermore, the physical half‐life (t_1/2_) of the nuclide needs to be on the one hand long enough for a work‐up procedure, processing and suitable for the time scale needed to track the biological process of interest, but on the other hand short enough to result in an acceptable radiation burden for the investigated subject. Last but not least, practical radionuclides need to be accessible.

Since these requirements are not easily fulfilled, only a relatively small number of radionuclides have made their way into clinical practice. For scintigraphy and SPECT applications, nuclides like indium‐111 (^111^In, t_1/2_ = 2.8 d), iodine‐123 (^123^I, t_1/2_ = 13.2 h), and technetium‐99m (^99m^Tc, t_1/2_ = 6.0 h) are nowadays routinely used, with ^99m^Tc being the working horse of nuclear medicine. For PET applications, an imaging technique based on the detection of annihilation radiation of positrons (β^+^), mainly short‐living nuclides produced by proton bombardment of appropriate targets in cyclotrons, are applied. Here, fluorine‐18 (^18^F, t_1/2_ = 109.7 min) has become the most common nuclide because of its accessibility and excellent physical properties. Other important PET nuclides in this context are carbon‐11 (^11^C, t_1/2_ = 20.4 min), nitrogen‐13 (^13^N, t_1/2_ = 10.0 min), and oxygen‐15 (^15^O, t_1/2_ = 2.0 min). Furthermore, gallium‐68 (^68^Ga, t_1/2_ = 67.6 min) has increasingly found applications as a PET radiometal because of the introduction of ^68^Ga generators and the establishment of somatostatin‐ and prostate‐specific membrane antigen (PSMA) tracers in the clinic.^2^ However, all these PET nuclides have a t_1/2_ of 2 to 110 minutes. Therefore, they are not suitable to track compounds *in vivo* that exhibit a slow pharmacokinetic (several hours to days), such as antibodies, cells, nanoparticles, and liposomes.

The number of applications of antibodies radiolabelled with a PET radionuclide (immunoPET) has significantly increased over the last decade.^3^ Consequently, PET nuclides with a longer t_1/2_ like zirconium‐89 (^89^Zr, t_1/2_ = 78.4 h) or copper‐64 (^64^Cu, t_1/2_ = 12.7 h) have been investigated to match an antibody's biological half‐life.^4^ As of recently, the PET isotope manganese‐52g (^52g^Mn) has been proposed as a suitable candidate for the combination with antibodies and proteins. It can be readily produced with a standard 16‐MeV cyclotron and has the potential to be used in bimodal PET/magnetic resonance (MR) systems as traceable nuclide in combination with a paramagnetic contrast agent based on nonradioactive manganese at the same time.

In this review, we give an overview of medically relevant β^+^ emitting manganese isotopes and their utility for applications in PET and PET/MR imaging to address questions of medical relevance. Although publications about the preclinical use of [^51^Mn]MnCl_2_ are available, we mainly focus on the longer‐living ^52g^Mn compounds and their applications.

## 2 RELEVANT PET RADIONUCLIDES OF MANGANESE FOR MEDICAL IMAGING

### 2.1 Physical properties

Table 1 provides an overview of all neutron‐deficient manganese isotopes with a t_1/2_ useful for medical applications (>1 min). Manganese‐53 (^53^Mn) and ‐54 (^54^Mn) are excluded from the discussion below because of their overall poor physical decay characteristics, such as negligible β^+^ intensity and unfavourably long t_1/2_. However, they have to be considered as possible side products/contaminants during the production of other manganese isotopes.

**Table 1:** Positron emission tomography (PET) isotopes of manganese and their physical properties

Isotope	ß^+^, %	E_ßmax_, keV	I_γ_, keV^a^	Half‐life
^51^Mn	97	2185.27	–	46.2 min
^52m^Mn	97	2633.36	1434.07 (98.3%)	21.1 min
^52g^Mn	29	575	744.23 (90.0%); 935.54 (94.5%); 1434.07 (100%)	5.6 d
^53^Mn	0/EC	–	X‐rays only	3.74 × 10^6^ y
^54^Mn	5.7 × 10^−7^	1377	834.85 (99.9%)	312.2 d

a Most abundant (intensity > 10%).

^51^Mn has a favourable β^+^ branching fraction and a t_1/2_ (t_1/2_ = 46 min) comparable with that of ^68^Ga (t_1/2_ = 68 min), which is suitable for the imaging of fast biological processes. However, the short t_1/2_ of ^51^Mn leads to constraints regarding target separation and radiolabelling chemistry. The β^+^ energy is relatively high (E_ßmax_ = 2.19 MeV) in comparison with the “standard” PET nuclide ^18^F (E_ßmax_ = 0.6 MeV), which leads to an unfavourably long penetration range of the β^+^s in tissue and, thus, a deteriorated spatial resolution in the PET images.^5^

^52m^Mn has a shorter t_1/2_ than ^51^Mn, comparable with that of the widely used ^11^C (t_1/2_ = 20 min), which renders its radiochemical handling even more challenging. Furthermore, the β^+^ energy of ^52m^Mn (2633 keV) is higher than that of ^51^Mn. This results in a mean ß^+^ range of 5.3 mm in tissue^6^ and, thus, in a correspondingly lower resolution of PET images. Additionally, ^52m^Mn decays partially via internal conversion to its ground state ^52g^Mn, leading to an increasing contamination with the longer‐living ^52g^Mn over time. A further drawback of this isotope with regard to medical applications is the presence of an additional prompt γ with relatively high energy (Table 1). Together with the β^+^ branching fraction of 93%, the arising dose rates and radiation protection concerns are unfavourable.

In contrast to the two Mn isotopes discussed above, ^52g^Mn has a convenient long t_1/2_ (5.6 d), which is advantageous for target separations and chemical handling of the radionuclide. In addition, its t_1/2_ is well suited for the investigation of slow biological processes, eg, the pharmacokinetics of antibodies. ^52g^Mn decays with a branching fraction for β^+^ of 29%, which is significantly lower than that of the previously mentioned Mn isotopes (Table 1). The β^+^ are emitted with a low maximum energy of E_ßmax_ = 0.6 MeV, which is among the lowest energies of all β^+^ emitting nuclides. This results in a comparatively low tissue penetration range of the β^+^s and, thus, better resolution of PET images. The main disadvantage of ^52g^Mn is the occurrence of three prompt γ rays with high energy and intensity (Table 1). These γ rays would significantly contribute to the radiation burden of patients and personnel of nuclear medicine departments. Furthermore, the prompt gammas cause erroneous signals in the PET detectors, which necessitates the implementation of prompt gamma correction techniques when using ^52g^Mn for PET imaging.^7^

### 2.2 Nuclide production

In theory, a large number of nuclear reactions can lead to the formation of each of the different manganese isotopes discussed in this review, including possible side products such as ^53^Mn and ^54^Mn (see IAEAs EXFOR database).^8^ However, many of the reported production routes have no practical relevance, and a comprehensive discussion of all possibilities is beyond the scope of this review. Instead, we focus on the most efficient ones, which includes the irradiation of solid chromium targets.

The production of ^51^Mn was discussed by Klein et al including a survey of potential nuclear reactions as well as a short summary of previous results by other groups.^9^ They concluded that the ^50^Cr(d,n)^51^Mn reaction previously investigated by Cogneau et al^10^ and the ^nat^Cr(p,x)^51^Mn reactions are the most promising candidates for this task. The ^50^Cr(d,n)^51^Mn reaction is based on the irradiation of isotopically enriched ^50^Cr with 14 → 3 MeV deuterons, resulting in the desired isotope in good yields (Table 2). The alternative reaction ^nat^Cr(p,xn)^51^Mn utilizes the high abundance of ^52^Cr (83.8%) in natural chromium and uses the ^52^Cr(p,2n)^51^Mn reaction. However, even if conducted with an optimized proton energy window, the coformation of the long‐living ^54^Mn and ^52m^Mn as well as ^52g^Mn by competing (p,n) reactions cannot be avoided. It should be mentioned that the formation of ^54^Mn can be circumvented by using highly enriched ^52^Cr as target material. The potential formation of long‐living ^53^Mn when using enriched ^52^Cr targets has not been investigated so far.

**Table 2:** Positron emission tomography (PET) isotopes of manganese and their production routes

Isotope	Reaction Channel	Energy Threshold, MeV^a^	Thick Target Yield, MBq/μAh
^51^Mn	^52^Cr(p,2n)^51^Mn^a^	16.3	Not determined
	^53^Cr(p,3n)^51^Mn^a^	24.4	Not determined
	^50^Cr(d,n)^51^Mn^b^	0	110^b^
^52m^Mn	^52^Cr(p,n)^52m^Mn	5.9	6910 ± 760
	^53^Cr(p,2n)^52m^Mn	13.8	
^52g^Mn	^52^Cr(p,n)^52g^Mn	5.5	13.7 ± 1.6
	^53^Cr(p,2n)^52g^Mn	13.4	

a Energy range 16.9 → 8.2 MeV protons for the nuclear reaction ^nat^Cr(p,xn).

b Energy range 14 → 3 MeV deuterons for the nuclear reaction ^50^Cr(d,n) with enriched target material.^9^, ^11^

One of the most promising routes for the production of ^52g^Mn and ^52m^Mn is represented by the irradiation of suitable chromium targets with 16 → 8 MeV protons (Table 2). Several studies demonstrated the feasibility of ^52^Mn production by the ^nat^Cr(p,xn)^52^Mn reaction using 16 MeV cyclotrons.^12^, ^13^, ^14^ Another study presents cross‐sectional data for target beams up to 20 MeV.^15^ Because of similar energy thresholds, ^52m^Mn is always coproduced with ^52g^Mn when chromium targets are irradiated with protons. An isomerically pure production of these nuclides is therefore not possible by this approach. However, for the production of ^52g^Mn for imaging applications, this is not an issue. Because of the significant difference in t_1/2_ of ^52g^Mn (21.1 min) and ^52m^Mn (5.6 d), the contamination can be removed by just simply letting the ^52m^Mn decay. The same applies also to potentially coproduced ^51^Mn. The only reported relevant impurity of ^52g^Mn mentioned in the literature is ^54^Mn, which is produced in minor amounts from ^54^Cr by a (p,n) reaction. Information about the potential long‐living impurity ^53^Mn is scarcely discussed albeit considerable cross sections for the ^53^Cr(p,n)^53^Mn reaction have been published.^16^ However, the formation of both long‐living impurities (^53^Mn and ^54^Mn) can be avoided by irradiation of highly enriched ^52^Cr and should therefore not impose any restriction for potential clinical applications of ^52g^Mn.

Alternatively to the production of Mn isotopes via proton irradiation, ^52m^Mn is available in high isotopic purity via a ^52^Fe/^52m^Mn generator (t_1/2_(^52^Fe) = 8.28 h).^17^ However, because of the poor accessibility of the mother nuclide ^52^Fe, this approach has not been fully explored yet.

### 2.3 Target separation

To separate ^52g^Mn isotopes from the chromium target material, several chromatographic ion exchange methods have been published.^18^, ^19^, ^20^ In most of them, the chromium target disc is first dissolved in an acidic medium and flushed over an ion exchange column. The radiomanganese is retained while chromium is washed out of the column. In a next step, the radiomanganese is eluted from the column with a different solvent composition. In the case of insufficient removal of chromium, the procedure has to be repeated. Different ion exchange resins, solvents, and solvent mixtures have been evaluated. For example, a recently published method required multiple subsequent column purifications resulting in a ^52g^Mn recovery below 70%.^21^ Thus, there is still room for future improvements. A further development is the combination of chemical and chromatographic separation techniques, resulting in higher purity of the desired ^52g^Mn.^22^ The different purification conditions are summarized in a review by Chaple et al,^18^ although none of them can be considered yet as “perfect” in terms of simplicity and recovery of the radionuclide.

It should be noted that, unlike in case of ^52g^Mn, these elaborate and lengthy separation techniques are not adequate to isolate the short‐living manganese PET isotopes ^52m^Mn and ^51^Mn, if produced via a solid chromium target. The development of a suitable liquid target enabling fast separation by solid phase extraction technology might provide a solution (a similar discussion is ongoing regarding the cyclotron production of ^68^Ga).^23^

In summary, the transition metal manganese offers three isotopes interesting for potential PET applications. Two of those, namely, ^51^Mn and ^52m^Mn, are short living and thus, have the potential for the imaging of fast biological processes. In comparison, ^51^Mn is the better candidate because of its favourable decay characteristics (longer t_1/2_, practically no additional γ‐rays), which are comparable with those of ^68^Ga. ^52g^Mn, on the other hand, has a suitable half‐life for the PET imaging of slow biological processes including applications in immunoPET. However, the presence of several high‐energy γ rays with high intensity necessitates the use of suitable corrections in PET imaging and is demanding with regard to radiation protection.

## 3 MANGANESE‐52G IN PRECLINICAL RESEARCH

### 3.1 Manganese complexes—coordination behaviour and paramagnetism

**Figure 1:**
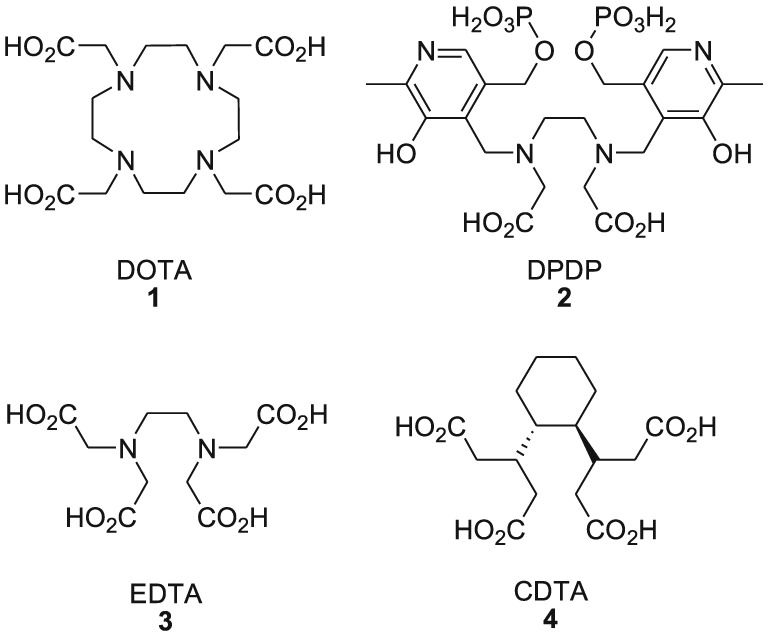
Examples of important chelating agents for manganese (II)

Manganese is a first‐row transition metal and homologue of technetium and rhenium. A broad range of coordination compounds of manganese are known, most of them with the metal in the oxidation state +II and coordination numbers of 6 or 7.^24^ As a “hard” Lewis acid, the most stable manganese complexes are obtained with ligands coordinating via oxygen and nitrogen atoms. For example, manganese (II) forms stable complexes with DOTA **1** and DO3A (logK_ML_ = 19.89 and 19.30, respectively).^25^, ^26^, ^27^ Such complexes can be obtained at room temperature under mild reaction conditions and within short reaction times.^25^ Other studied chelators for the complexation of Mn (II) are EDTA **3** and its derivatives such as CDTA **4** (Figure 1).^28^

**Figure 2:**
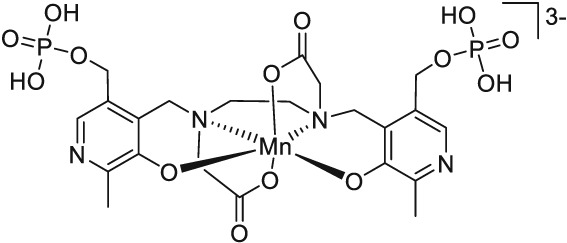
Mn‐DPDP (Teslascan), a manganese‐based T_1_ contrast agent

Mn (II) in its octahedral high spin complexes owns five unpaired electrons, which results in a high paramagnetic moment. For this reason, manganese (II) complexes have been investigated as possible magnetic resonance imaging (MRI) contrast agents. For example, the dipyridoxyl diphosphate (DPDP) complex of manganese (II) (Teslascan, Figure 2) was used in the clinic for the diagnosis of liver lesions. However, the compound has meanwhile been withdrawn from the market because of its insufficient stability in vivo.^29^

### 3.2 Biological pathways of MnCl_2_ in vivo and neuronal connectivity imaging

Manganese is an essential element for all beings, for example, participating in numerous enzymatic processes as a cofactor.^30^ In the oxidation states +II and +III, it is transported in the blood bound to serum proteins^31^ before it accumulates in organs and tissue or is excreted.^32^ Each human body contains roughly 12 mg of manganese mostly stored in the bones, liver, and kidney.^33^ Notably, it is also known to cross the blood‐brain‐barrier.^34^ In higher doses, free manganese ions are known to cause a neurological disorder condition called manganism with psychiatric and, in later stages, Parkinson‐like symptoms.^35^

With regard to potential medical applications of manganese compounds, the pharmacokinetic profile of intravenously injected [^52g^Mn]MnCl_2_ was investigated in mice.^36^ The highest accumulation of the radiometal was found in the liver, kidney, salivary glands, thyroid, and pancreas, whereas only low uptake of radioactivity was observed in the bones. These data may help to predict the fate of Mn (II) ions in case they get released from radiopharmaceuticals in vivo. Interestingly, the biodistribution pattern changed when [^52g^Mn]MnCl_2_ was given by inhalation as a saline aerosol (eg, decreased uptake in the bones).^36^

To study the uptake and retention of manganese with regard to possible neuroimaging techniques, manganese‐enhanced magnetic imaging (MEMRI) as well as PET imaging was utilized by Brunnquell et al.^37^ For this purpose, different doses of nonradioactive MnCl_2_ were administered intravenously to rats. The uptake in different parts of the brain was studied using quantitative MEMRI at different time points postinjection (24 h to 14 d). Brain uptake studies with nca and ca [^52g^Mn]MnCl_2_ were performed via gamma counting of resected brain areas. However, the authors concluded that the brain uptake of [^52g^Mn]Mn^2+^ with intact blood‐brain barrier was too low for neuroimaging applications, especially when using the carrier added radiotracer. In addition, the authors stated that [^52g^Mn]MnCl_2_, although not suitable in this case, still holds promise as a radiotracer for specific uptake in the salivary glands and pancreas. Another publication evaluated ^52g^Mn for neuroimaging by stereotactic injection into the rat brain.^38^ The neuronal pathways between rat brain regions could be imaged successfully. Through application of different doses of nca [^52g^Mn]MnCl_2_ (30 kBq‐170 MBq), the radiotoxicity was also studied, revealing that a low dose of 20 kBq is sufficient for imaging, while no histological and behavioural noxious effects occurred.

Saar et al studied the biodistribution of ^51^Mn and ^52g^Mn in different organs.^39^ Further, they investigated the neuronal olfactory pathway in monkeys and rodents using nasally administrated ^51^Mn and ^52g^Mn. It was shown that an administration of [^52g^Mn]MnCl_2_ allows for a tracing of neuronal connections and that ^52g^Mn is able to enter excitable cells in a similar manner as nonradioactive manganese ions in MEMRI.

### 3.3 Manganese‐52g for immunoPET, cell labelling, and ß‐cell mass monitoring

The use of radiolabelled antibodies for immunoPET has become an important tool in nuclear oncology.^3^, ^40^ Radiolabelled antibodies offer the advantage of high affinity and specificity. On the other hand, antibodies exhibit slow pharmacokinetics and the time until satisfying tumour uptake and/or tumour‐to‐background ratios are reached is often too long for other short‐living PET radionuclides such as ^68^Ga, ^18^F, or even ^64^Cu. Therefore, the longer‐living ^89^Zr (t_1/2_ = 78.4 h) is currently predominantly used for the labelling of clinically relevant antibodies.^40^ However, ^52g^Mn also offers a suitable t_1/2_ for immunoPET applications while displaying a lower β^+^ energy and a higher β^+^ intensity than ^89^Zr (Table 3). Thus, ^52g^Mn may represent a promising, alternative radiometal for applications in immunoPET.^41^

**Table 3:** Nuclides used for radiolabelling of antibodies and their physical properties

Nuclide	ß^+^, %	E_ßmax_, keV	I_γ_, keV^a^	Half‐life, h
^64^Cu	17.9	653	–	12.7
^89^Zr	22.8	902	909 (99.0%)	78.4
^52g^Mn	29	575	744.23 (90.0%); 935.54 (94.5%); 1434.07 (100%)	134.4

a Most abundant (intensity > 10%).

**Figure 3:**
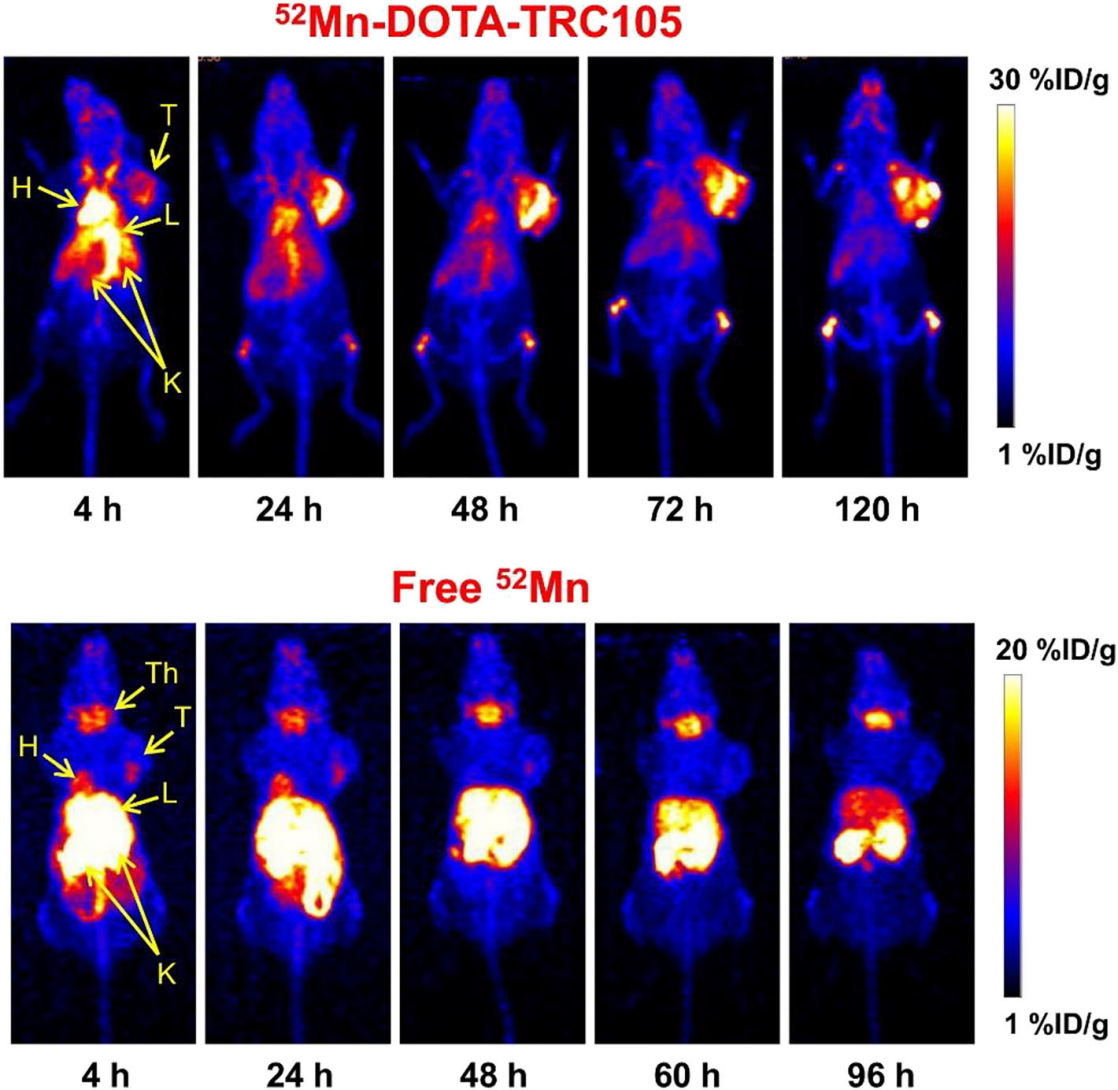
Serial maximum intensity projection (MIP) positron emission tomography (PET) images of mice injected with ^52^Mn‐DOTA‐TRC105 and ^52^MnCl_2_. Significant thyroid accumulation in the ^52^MnCl_2_ images contrasting the lack of uptake in the ^52^Mn‐DOTA‐TRC105 images indicates highly stable DOTA chelation of ^52^Mn^2+^ even at late time points. H, heart; K, kidneys; L, liver; T, tumour; Th, thyroid. Reprinted with permission from Graves et al^21^

The first and so far only in vivo study with a ^52g^Mn‐labelled antibody was published in 2015 by Graves et al.^21^ For this study, the chelator DOTA was conjugated to the AT1‐targeting antibody TRC105 and tested in radiolabelled form for the PET imaging of a breast cancer xenograft mouse model. The chelator DOTA allowed for the ^52g^Mn‐labelling at room temperature (see above), which is a necessary requirement to avoid denaturation of the protein during radiolabelling. Despite a slower blood clearance, the in vivo biodistribution and PET imaging yielded comparable results to a similar ^89^Zr‐labelled antibody conjugate (Figure 3). The research group used ^52g^Mn instead of the more common ^89^Zr to demonstrate the opportunity of PET imaging at late time points postinjection of the radiotracer and the possibility for triple coincidence PET measurements,^42^ a new imaging technology for which ^52g^Mn has suitable physical properties.

Another potential use for longer‐living radiometals in nuclear medicine is the labelling and tracking of cells and liposomes in vivo. In 2018, Gawne et al demonstrated the suitability of [^52g^Mn]Mn (oxinate)_2_ to label different cell types as well as DOXIL/CAEXYL liposomes.^43^ It was shown that the efficiency of the radiolabelling was comparable with analogous ^89^Zr‐labelling, although the method was limited by cell efflux of [^52g^Mn]Mn^2+^. In vivo studies of ^52g^Mn‐labelled liposomes in mice showed sufficient stability of the conjugate for up to 24 hours as long as the compound remained in the bloodstream. After the liposomes entered cells and tissues, uptake of radioactivity in the kidneys, salivary glands, and pancreas was detected, indicating decomposition and release of free [^52g^Mn]Mn^2+^ ions. The authors concluded that their method was not suitable for in vivo tracking of cells but could serve as a model to study the biological fate of ^52g^Mn once delivered inside of cells in vivo.

A different approach towards radiolabelled liposomes using ^52g^Mn was published by Jensen et al.^44^ In this comparative study, ^52g^Mn(II) and ^64^Cu(II) were prepared as their DOTA chelates and used for both internal loading and surface labelling of liposomes. In vivo biodistribution studies with the ^52g^Mn‐labelled liposome preparations revealed that liposomes with internal ^52g^Mn‐loading had a longer plasma half‐life than their surface labelled counterparts. The authors concluded that the reduced blood plasma half‐live of the surface modified liposomes could result from insufficient in vivo stability of the radiometal‐DOTA chelates.^44^

Mn^+2^ was shown to be taken up significantly by the pancreas^31^ (see also above), possibly by mimicking Ca^2+^ in pancreatic metabolic pathways. This feature was used by Hernandez et al to monitor ß‐cell mass with [^52g^Mn]MnCl_2_ by ex vivo and in vivo imaging of ß‐cell metabolism in type 1 and type 2 diabetes mouse models.^45^ Previous work on this topic using MRI and nonradioactive manganese ions as contrast agents showed that the utility of this method was limited by the toxicity of free Mn^2+^ ions. Using the radiotracer [^52g^Mn]MnCl_2_ and PET imaging solved the toxicity issues. It was shown that the uptake of [^52g^Mn]Mn^2+^ strongly depends on the activity of ß‐cell voltage‐dependent Ca^2+^ channels and that the uptake of the radiometal correlates with Ca^2+^ uptake. The authors concluded further that because of the rapid uptake mechanisms, the use of the shorter‐living ^51^Mn might be a good alternative. This was later confirmed by a subsequent study using [^51^Mn]MnCl_2_.^46^

### 3.4 Relaxivity and stability—manganese for PET/MRI

In the past years, complexes of paramagnetic Mn (II) have been discussed as potential alternatives to the well‐established, but in some cases, disputed gadolinium‐based contrast agents (eg, Gadopentetat‐Dimeglumine, Magnevist).^47^, ^48^ For details on Mn‐based MRI contrast agents, the reader is referred to an excellent review on the topic.^49^ In addition, the availability of PET nuclides of manganese offers the possibility of isotopically radiolabelled manganese MR contrast agents for use in bimodal PET/MR imaging. Hybrid imaging by PET/MRI has lately received considerable attention in the nuclear medicine and radiology because it combines the high sensitivity PET with the high resolution of MRI.^50^ The concept is attractive, however, hampered by the different sensitivities of the modalities: PET allows for the application of very low concentrations of the radioactive substance (10^−9^‐10^−12^M) for achieving excellent contrast. In comparison, paramagnetic contrast agents for MRI are applied in millimolar concentrations (one dose of Gd‐DTPA, Gadovist: 0.1 mmol/kg body weight).^51^ The contradicting requirements of the two modalities for contrast agents can be met by using mixtures of a PET tracers with the respective nonradioactive analogous MRI contrast agent (carrier added radiotracers). Because the PET tracer (^52g^Mn) and the MR contrast agent (^nat^Mn) are structurally identical, they exhibit equal biological properties.^52^ Employment of such mixtures enables, eg, the quantification of the MR contrast agent in areas of low uptake by PET. Furthermore, the concept is particularly attractive for applications to “smart MR contrast agents”.^53^, ^54^

The ability of a coordination compound to enhance longitudinal MR contrast is described by the value “relaxivity” r_1_. The relaxivity of paramagnetic coordination compounds is influenced not only by the number of unpaired electrons of the central metal ion but also by its internal rotation, size, and most importantly, magnetic influence on directly bound and surrounding water molecules.^55^

Since a water molecule as an additional ligand inside the coordination sphere of the metal is required for contrast enhancement, Mn (II) complexes with hexa‐ or lower dentate ligand systems and at least one inner sphere water molecule are potential candidates for MRI contrast agents.^60^ The tendency of the chelator DOTA **1** to form octadentate complexes with manganese of excellent stability (see above) has its downside in this context: Because of the lack of an inner sphere water molecule in [^nat^Mn (DOTA)], there is no MR contrast (Table 4). Therefore, complexes of Mn (II) with DOTA **1** (and DO3A)^28^ have good properties for PET imaging but are not suitable for PET/MR applications.

**Table 4:** Contrast agents based on Gd and Mn and their physicochemical properties

Contrast Agent	Longitudinal Relaxivity r_1_, 37°C, 20 MHz [mmol^−1^s^−1^]	Stability Constant logK_ML_ a	Ref
Gd‐DOTA (Dotarem)	3.83	24.7	56, 57, 58
Gd‐DTPA (Magnevist)	4.02	22.5	25
Mn‐DPDP (Teslascan)	2.80	11.6	59
Mn‐CDTA	2.75	14.3	28
Mn‐PyC3A	2.10	14.1	60
Mn‐DOTA	–	19.9	60
Mn‐DO3A	1.30	19.4	27

a K_ML_ = Equilibrium stability constant: [ML]/[M]^*^[L] with [ML] complex concentration; [M] metal ion concentration; [L] ligand concentration, in equilibrium.

**Figure 4:**
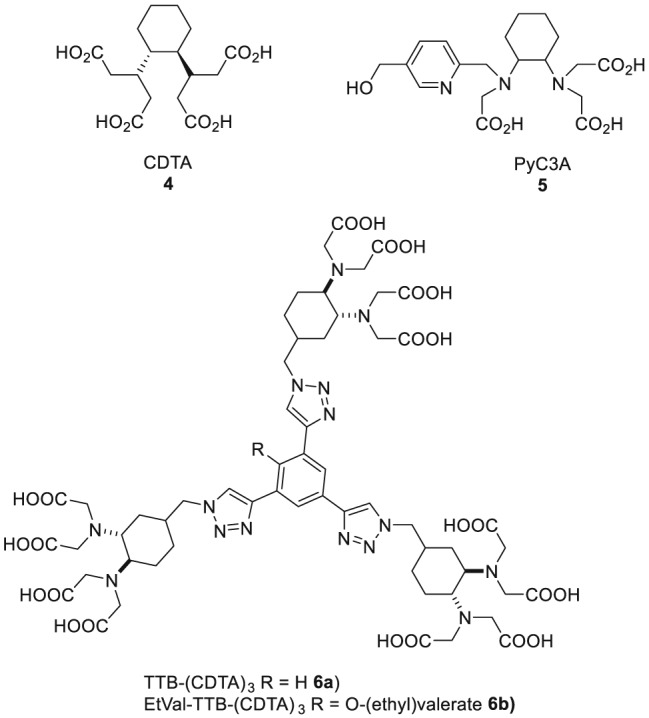
Successful chelating agents for Mn based on CDTA **4** for magnetic resonance imaging (MRI) using manganese

While the combination of Mn (II) with DOTA **1** provides stable complexes but no contrast enhancement, the use of other chelators (eg, EDTA **3**) results in complexes with appropriate relaxivity but insufficient stability. Therefore, the quest for new chelators that fulfil both requirements is still ongoing. The best compromise between sufficient stability and contrast enhancement described so far was achieved with chelators on the basis of the 1,2‐trans‐cyclohexyldiaminocarboxylate (CDTA **4**) scaffold, for example, PyC3A **5** (Figure 4 with structure **4**‐**6**).^56^ Gale et al reported in 2015 that the Mn (II) complex of PyC3A proved to be of sufficient thermodynamic stability (logK_MnL_ = 14.14) and suitable for contrast‐enhanced MR angiography.^60^ This successful proof‐of‐concept study represents an important step towards the development of new alternatives of Gd‐based MR contrast agents.

In 2016, Vanasschen et al published the first isotopically labelled bimodal PET/MRI agent based on mixtures of radioactive and nonradioactive manganese.^61^ The stability of the investigated [^52g/55^Mn]Mn‐CDTA complex in human blood serum (HBS) was studied, as well as its radiolabelling reaction using nca and ca [^52g/55^Mn]Mn^+2^. The radiolabelling could be performed successfully (radiochemical yield > 99%) at room temperature within 30 minutes. The stability study in HBS showed a dissociation half‐life of [^52g/55^Mn]Mn‐CDTA of 12 hours with most of the released [^52g^Mn]Mn^2+^ being bound to larger serum proteins.^61^

Inspired by earlier work of Zhu et al,^62^ a rigid dendrimeric scaffold with three isotopically ^52g/55^Mn‐labelled complexes has been recently reported.^63^ The investigated contrast agent ^nat^Mn‐Tris‐CDTA‐1,3,5‐tris‐triazolobenzene [^nat^Mn][Mn_3_(TTB‐(CDTA)_3_] **6a** exhibited a dramatically increased overall T_1_ relaxivity in comparison with monomeric Mn‐CDTA, obviously the result of the presence of multiple paramagnetic centres. Also, the relaxivity of each paramagnetic centre could be increased by 144% because of the rigidity and restricted internal rotation of the molecule in comparison with Mn‐CDTA.^28^, ^63^ Labelled with isotopic mixtures of ^nat^Mn and ^52g^Mn, a bimodal PET/MR contrast agent based on manganese with high relaxivity was obtained. Through functionalization of the chelator, a bifunctionalized chelating agent ((EtVal‐TTB‐(CDTA)_3_ **6b** was synthesized to be used for further derivatization and potentially for bioconjugations.

It should be noted that other approaches for the development of PET/MRI imaging agents have also been reported. For example, Notni et al combined [^68/69^Ga]Ga and ^nat^Gd in a scaffold containing the chelators TRAP and DOTA^52^; Frullano et al studied ^nat^Gd‐DOTA complexes with a pendant ^18^F atom as PET reporter.^53^ In addition, iron particles spiked with radionuclides have been studied by different groups as potential PET/MRI imaging agents.^64^

## 4 SUMMARY AND CONCLUSION

There are three isotopes of manganese that are interesting for PET applications. The most promising among them is ^52g^Mn due to its low β^+^ energy and suitable t_1/2_. ^52^Mn can be readily produced using a small 16‐MeV cyclotron and separated from the target material by effective but time‐consuming ion exchange chromatography. For clinical applications, ^52g^Mn with a half‐life of 5.5 days is an interesting candidate for the radiolabelling of molecules with slow pharmacokinetics, for example, antibodies for immunoPET. In this context, ^52g^Mn outperforms the current standard for immunoPET ^89^Zr not only in terms of t_1/2_ but also in terms of β^+^ energy, the latter resulting in better resolution of PET images for ^52g^Mn. On the other hand, a drawback of ^52g^Mn in comparison with ^89^Zr is the occurrence of high‐energy γs that will increase the dose rates for the patients, as well as the lack of commercial sources, which currently restricts its use in radiopharmaceutical research.

Because ^nat^Mn (II) is paramagnetic and PET isotopes of the metal are available, isotopically radiolabelled manganese‐based PET/MRI contrast agents are within reach. The main challenge is the identification of suitable chelators, which provide manganese complexes of sufficient stability and relaxivity. Different approaches towards the development of such manganese complexes have been reported, but issues regarding the in vivo stability remain to be addressed.

In summary, manganese is a versatile transition metal with available isotopes well suited for MR, PET, and PET/MR imaging. Because of the currently limited availability, only a relatively small number of publications describe the production and use of manganese PET radionuclides. However, this may change in the future as in particular ^52g^Mn has high potential to become a new emerging radiometal for PET and PET/MR applications in nuclear medicine.
